# Association of Presepsis Statin Prescription With Kidney and Mortality Outcomes: Cause-Specific, Overlap-Weighted Analyses

**DOI:** 10.1016/j.xkme.2026.101397

**Published:** 2026-05-08

**Authors:** Min Woo Kang, Soojeong Yun, Seung Min Song, Ji Eun Kim, Hyo Jin Kim, Eun Jung Cho, Young Joo Kwon, Shin Young Ahn

**Affiliations:** 1 Department of Internal Medicine, Korea University Guro Hospital, Seoul, Republic of Korea; 2 Department of Internal Medicine, Korea University College of Medicine, Seoul, Republic of Korea

**Keywords:** kidney injury, mortality, sepsis, statin

## Abstract

**Rationale & Objective:**

Sepsis, complicated by kidney injury, poses a mortality risk in intensive care unit patients. Although statins are thought to offer protective effects against kidney injury through anti-inflammatory mechanisms, evidence remains inconclusive. This study aimed to evaluate whether statin prescription before sepsis onset affects the risks of kidney injury and mortality.

**Study Design:**

Retrospective cohort study.

**Setting & Participants:**

Adult intensive care unit patients who met Sepsis-3 criteria in Medical Information Mart for Intensive Care-IV.

**Exposure:**

Statin prescription within the 24 hours preceding sepsis onset.

**Outcomes:**

The primary outcome was the prespecified kidney outcome (need for kidney replacement therapy or a reduction in estimated glomerular filtration rate of ≥50%). Secondary outcomes were mortality without the kidney outcome (cause-specific) and overall mortality.

**Analytical Approach:**

We used overlap weighting to balance baseline characteristics. Cause-specific Cox models estimated associations for the kidney outcome and mortality without the kidney outcome; a weighted Cox model estimated associations for overall mortality.

**Results:**

The final cohort included 30,765 patients with sepsis, with 19.3% exposed to statins. Statin prescription was associated with a reduced risk of kidney outcome (HR, 0.83; 95% CI, 0.80-0.87), mortality without kidney outcome (HR, 0.56; 95% CI, 0.51-0.63), and overall mortality (HR, 0.78; 95% CI, 0.71-0.84). Subgroup analyses were broadly consistent across prespecified strata. For the kidney outcome, interaction testing suggested greater risk reduction among patients aged <65 years, those without chronic kidney disease, and those with hypertension. For death without prior kidney outcome and for overall mortality, protective associations were consistent across subgroups, with a larger effect among patients with prior myocardial infarction. Overall, statin prescription was consistently associated with lower risks of kidney outcome and mortality.

**Limitations:**

Single-database retrospective design and incomplete capture of sepsis severity measures.

**Conclusions:**

Presepsis statin prescription was associated with lower risks of kidney outcome and mortality, indicating potential clinical benefits in patients with sepsis.

## Plain-Language Summary

Sepsis is a severe infection that can threaten life and damage organs. Statins are medicines best known for lowering cholesterol, but they may also reduce inflammation and stabilize blood vessels. We studied adults in intensive care who met modern (Sepsis-3) criteria for sepsis and compared patients who were prescribed a statin within 24 hours before sepsis onset with those who were not. People who were taking statins before sepsis started had lower risks of kidney function decline and death. The association seemed stronger for survival than for kidney outcomes. These findings suggest that statins may offer clinical benefits for patients with sepsis and motivate future work to determine which statin types and doses are most helpful.

Sepsis is a condition associated with exceptionally high mortality rates in the intensive care unit (ICU), with acute kidney injury (AKI) occurring in approximately 40%-50% of patients with sepsis.^1^^,^^2^ Mortality among patients with sepsis who develop AKI can reach up to 41% in ICU settings, significantly exceeding mortality rates in those without AKI.^3^, ^4^, ^5^, ^6^ As AKI is a major risk factor that worsens prognosis in sepsis, particularly due to the severe inflammatory responses involved in its pathogenesis, the need for treatments to prevent or reduce AKI in patients with sepsis is urgent. However, specific pharmacologic agents effective in preventing or mitigating AKI in sepsis patients remain largely unidentified. Thus, developing therapeutic strategies that can reduce not only mortality but also AKI in this high-risk patient population is crucial.

Previous studies suggest that statins may have protective effects against AKI through their anti-inflammatory and vascular protective properties. Statin use, for example, has been associated with reduced AKI incidence after cardiac surgery and with the prevention of contrast-induced AKI.^7^^,^^8^ Although an animal study indicates that statins may lower the risk of septic AKI, their effectiveness in humans has yet to be fully validated.^9^ Retrospective studies have also shown a trend toward reduced mortality in patients with sepsis treated with statins, possibly due to their anti-inflammatory effects and vascular protective mechanisms.^10^, ^11^, ^12^, ^13^, ^14^ A previous randomized controlled trial has demonstrated survival benefits associated with statin use in patients with severe sepsis.^15^ However, findings from meta-analyses and specific clinical trials have been inconsistent regarding the protective effects of statins in sepsis patients, underscoring the need for further research.^16^, ^17^, ^18^, ^19^

This study aimed to evaluate the impact of prior statin prescription before the onset of sepsis on kidney injury and mortality outcomes. Specifically, the research assessed whether statin administration preceding sepsis could provide protective effects on kidney function and independently reduce overall mortality, even in the absence of kidney injury. Utilizing the large-scale Medical Information Mart for Intensive Care (MIMIC)-IV dataset, the study employed cause-specific analysis to investigate the association between presepsis statin prescription and outcomes related to kidney injury and mortality in patients with sepsis.

## Methods

### Study Population

This study utilized data from the MIMIC-IV database, a large, publicly available dataset comprising deidentified ICU patient records from Beth Israel Deaconess Medical Center.^20^ MIMIC-IV version 3.0 was used, which includes data spanning from 2008 to 2022 and encompasses over 94,000 patient records with comprehensive information on demographics, vital signs, laboratory results, medications, procedures, and diagnoses. Mortality outcomes were ascertained using in-hospital death records and linked postdischarge vital status, which in MIMIC-IV enable follow-up of mortality for up to 1 year after hospital discharge.

Only patients admitted to the ICU with sepsis were included in the analysis. Sepsis was defined according to the Sepsis-3 criteria, which require an increase of ≥2 points in the Sequential Organ Failure Assessment score along with suspected or confirmed infection.^21^ Patients with underlying end-stage kidney disease or those receiving kidney replacement therapy at the time of sepsis diagnosis were excluded from the study.

### Ethical Approval and Consent to Participate Statement

The study was exempt from review by the Institutional Review Board of Korea University Guro Hospital (No. K2025-1397-001). As all data were anonymized and freely accessible, individual informed consent was not required.

### Exposure, Outcomes, and Variables

Exposure was defined by statin administration status, specifically considering patients as exposed if they had received a statin prescription within 24 hours before the sepsis diagnosis point, defined as the onset of sepsis with a ≥2-point increase in the Sequential Organ Failure Assessment score. The outcomes evaluated included kidney outcome, mortality without kidney outcome, and overall mortality. Kidney outcome was defined as initiation of kidney replacement therapy or a ≥2-fold increase in serum creatinine from baseline. Mortality was further divided into cases in which the kidney outcome did not occur (mortality without kidney outcome) and overall mortality. This division allowed for analysis of whether statins affected mortality independently of kidney outcome, given that kidney injury is a known risk factor for mortality in sepsis patients.^22^^,^^23^

Variables included demographic and baseline patient status data, such as age, sex, and weight at ICU admission. Comorbid conditions were defined based on *International Classification of Diseases* diagnostic codes documented at admission and included hypertension, diabetes, hypercholesterolemia, chronic kidney disease (CKD), myocardial infarction, congestive heart failure, cerebrovascular disease, and peripheral vascular disease. Additionally, initial values for systolic blood pressure, diastolic blood pressure, heart rate, fraction of inspired oxygen, peripheral oxygen saturation, and mechanical ventilator status were recorded at the time of sepsis diagnosis. Baseline estimated glomerular filtration rate (GFR) was defined as the highest estimated GFR recorded within the 180 days preceding sepsis onset among all recorded laboratory results. Missing values were handled using multiple imputation by chained equations.

### Cause-Specific Hazard Ratio for Outcomes With Overlap Weights

Since mortality precludes the occurrence of kidney outcomes, it is considered a competing risk for kidney outcome. Therefore, cause-specific analysis was conducted to calculate the cause-specific hazard ratio for both kidney outcome and mortality without kidney outcome.^24^ A Cox-based estimation model was used for this analysis, applying overlap weights estimated from a logistic regression.^25^ Additionally, to assess overall mortality risk, a multivariable Cox proportional hazard model with overlap weights was used, incorporating initial systolic blood pressure, diastolic blood pressure, heart rate, fraction of inspired oxygen, peripheral oxygen saturation, mechanical ventilator status, and baseline estimated GFR as covariates.

The variables included in the overlap weights were based on factors relevant to the indication for statin prescription and underlying cardiovascular risks.^26^, ^27^, ^28^, ^29^, ^30^, ^31^, ^32^, ^33^, ^34^, ^35^ In model 1, variables included age, sex, and weight; model 2 added hypertension, diabetes, hypercholesterolemia, and CKD to the variables in model 1; model 3 included additional cardiovascular comorbid conditions such as myocardial infarction, congestive heart failure, cerebrovascular disease, and peripheral vascular disease. Clinically, this set captures the principal indications for statin prescribing and baseline cardiovascular–kidney risk that plausibly confound both treatment assignment and outcomes, aligning with contemporary lipid management guidance^36^; kidney function reserve as a determinant of AKI and mortality risk in sepsis^2^; and acute illness severity at sepsis onset via hemodynamic and respiratory parameters, which also influence feasibility of oral therapy and prognosis.^21^^,^^37^ To evaluate the attained mean covariate balance after weighting, absolute standardized differences (ASDs) were calculated for each variable. Additionally, a cumulative incidence rate graph for each outcome based on model 3 was analyzed.

### Subgroup Analysis

Subgroup analyses were conducted across all outcomes, stratifying by age (≥65 years vs <65 years), CKD, hypercholesterolemia, diabetes, hypertension, and myocardial infarction. For kidney outcome and mortality without kidney outcome, cause-specific analyses were performed, whereas overall mortality was analyzed using a multivariable Cox proportional hazard model. Effect modification was evaluated by adding treatment-by-subgroup interaction terms; 2-sided *P* values for interaction were calculated. These analyses followed the same methodology as described previously, employing model 3 variables and applying overlap weights throughout.

### Sensitivity Analysis

For the sensitivity analysis, the same variables used in model 3 were applied, with stabilized average treatment effect (ATE) weighting used instead of overlap weighting. This approach was employed to reassess the risk associated with statin prescription across the 3 outcomes, ensuring robustness of the findings. We additionally fitted Fine–Gray subdistribution hazard models for kidney outcome and mortality using the model 3 covariate set with overlap weighting. To evaluate robustness to exposure timing and misclassification, we redefined statin exposure as any prescription within 7 days before sepsis onset and repeated the analytic workflow, estimating cause-specific hazards for kidney outcome and for death without prior kidney outcome, alongside a standard Cox model for all-cause mortality. Recognizing that the ability to receive oral therapy may confound associations, we selected a relatively common oral medication without a plausible kidney effect (oral laxatives) as a negative control exposure to probe bias related to oral administration feasibility and residual severity confounding, estimating associations via multinomial propensity scores with generalized overlap weights.

## Results

### Study Population and Baseline Characteristics

**Figure 1:**
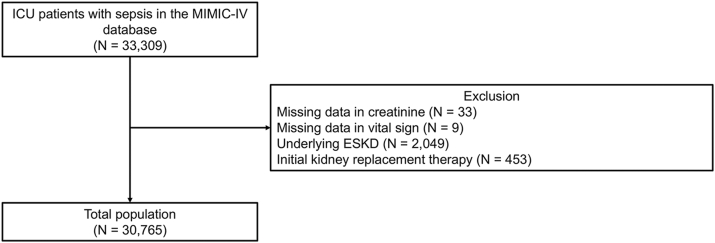
Flow diagram of the study population. Abbreviations: ESKD, end-stage kidney disease; ICU, intensive care unit; MIMIC-IV, Medical Information Mart for Intensive Care-IV.

From the MIMIC-IV dataset, a total of 33,309 patients met the Sepsis-3 criteria for sepsis. Among these, 33 patients without baseline creatinine values, 9 with missing vital sign measurements, 2,049 with underlying end-stage kidney disease, and 453 receiving kidney replacement therapy at the time of sepsis diagnosis were excluded, leaving a final cohort of 30,765 patients for analysis (Fig 1).

The mean age of the entire study population was 64.7 ± 16.0 years, with men comprising 57.6% (Table 1). Among the patients, 19.3% were treated with statins, whereas 80.7% were not. Hypercholesterolemia was present in 42.3% of the total population, with 35.4% in the non-statin group and 71.4% in the statin group. The median follow-up was 8.0 days (interquartile range, 4.9-14.5) (Table 2). All variables, except peripheral oxygen saturation, showed significant differences between the statin and non-statin groups. Only peripheral oxygen saturation had missing values (0.1%), which were imputed using multiple imputation by chained equations.

**Table 1:** Baseline Characteristics

Variable	Total Population (N = 30,765)	No Statin Prescription (n = 24,815)	Statin Prescription (n = 5,950)	ASD (Unweighted)	ASD (Overlap-Weighted)
Age (y)	64.7 ± 16.0	63.4 ± 16.6	70.3 ± 11.6	0.482	<0.001
Men	17,731 (57.6%)	13,976 (56.3%)	3,755 (63.1%)	0.139	<0.001
Weight (kg)	82.2 ± 25.5	81.7 ± 25.6	84.3 ± 24.9	0.102	<0.001
Systolic blood pressure (mm Hg)	94.8 ± 18.3	95.3 ± 18.5	92.8 ± 17.4	0.143	0.132
Diastolic blood pressure (mm Hg)	49.7 ± 11.8	50.3 ± 12.0	47.2 ± 10.5	0.279	0.160
Heart rate (/min)	100.5 ± 21.1	101.5 ± 21.5	96.4 ± 19.0	0.249	0.118
SpO_2_ (%)	93.5 ± 5.8	93.5 ± 5.8	93.5 ± 5.9	0.001	0.015
FiO_2_ (%)	46.1 ± 41.7	43.2 ± 40.6	58.22 ± 44.0	0.356	0.341
Mechanical ventilation	18,814 (61.2%)	14,765 (59.5%)	4,049 (68.1%)	0.179	0.087
eGFR (CKD-EPI) (mL/min/1.73 m^2^)	89.6 ± 31.0	91.1 ± 31.9	83.4 ± 26.2	0.264	0.065
Hypertension	15,358 (49.9%)	11,638 (46.9%)	3,720 (62.5%)	0.318	<0.001
Diabetes	9,658 (31.4%)	7,109 (28.6%)	2,549 (42.8%)	0.299	<0.001
Hypercholesterolemia	13,022 (42.3%)	8,776 (35.4%)	4,246 (71.4%)	0.774	<0.001
Chronic kidney disease	6,343 (20.6%)	4,710 (19.0%)	1,633 (27.4%)	0.202	<0.001
Myocardial infarct	5,401 (17.6%)	3,595 (14.5%)	1,806 (30.4%)	0.387	<0.001
Congestive heart failure	9,708 (31.6%)	7,119 (28.7%)	2,589 (43.5%)	0.312	<0.001
Cerebrovascular disease	4,660 (15.1%)	3,646 (14.7%)	1,014 (17.0%)	0.064	<0.001
Peripheral vascular disease	3,656 (11.9%)	2,622 (10.6%)	1,034 (17.4%)	0.197	<0.001

*Note:* Values are mean ± standard deviation or n (%).

Abbreviations: ASD, absolute standardized difference; CKD-EPI, Chronic Kidney Disease Epidemiology Collaboration; eGFR, estimated glomerular filtration rate; FiO_2_, fraction of inspired oxygen; SpO_2_, peripheral oxygen saturation.

**Table 2:** Follow-up Duration and Clinical Outcomes

Measure	Total Population (N = 30,765)	No Statin Prescription (n = 24,815)	Statin Prescription (n = 5,950)
Follow-up time (d)	8.0 (4.9-14.5)	8.3 (5.0-15.0)	6.8 (4.8-12.0)
Kidney outcome	11,933 (38.8%)	10,018 (40.4%)	1,915 (32.2%)
Mortality	4,978 (16.2%)	4,252 (17.1%)	726 (12.2%)

*Note:* Values are n (%) or median (interquartile range).

### Overlap and Stabilized ATE Weighting

The ASDs for each variable are presented in Fig S1 and Table 1. In the unadjusted analysis, imbalance was greatest for hypercholesterolemia and age. After applying overlap weighting, balance improved markedly across covariates, with residual imbalance confined mainly to acute physiologic measures—most notably fraction of inspired oxygen and to a lesser extent, blood pressure and heart rate—while ASDs for other variables were near zero. With stabilized ATE weighting, most variables did not exceed the standard ASD threshold of 0.1, although age and hypercholesterolemia exhibited the highest ASD values.^38^ For age and hypercholesterolemia, the 2 variables with the highest ASDs, distributions were compared across unadjusted, overlap weighting, and stabilized ATE weighting data (Fig S2). The unadjusted data showed minimal overlap between the distributions of these variables, while overlap weighting achieved the greatest alignment across both age and hypercholesterolemia.

### Hazard Ratio for Outcomes

**Figure 2:**
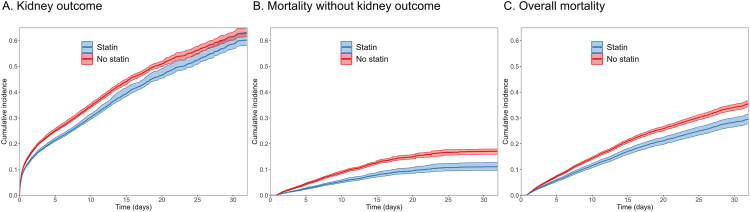
Cumulative incidence of outcomes. (A) Kidney outcome. (B) Mortality without kidney outcome. (C) Overall mortality.

Hazard ratios for each outcome associated with statin prescription are shown in Table 3. For all outcomes—kidney outcome, mortality without kidney outcome, and overall mortality—statin prescription was associated with a statistically significant reduction in risk across the unadjusted model and models 1, 2, and 3. In model 3 specifically, statin prescription reduced the risk of kidney outcome, mortality without kidney outcome, and overall mortality with hazard ratios of 0.83 (95% confidence interval [CI], 0.80-0.87), 0.56 (95% CI, 0.51-0.63), and 0.78 (95% CI, 0.71-0.84), respectively. The cumulative incidence rates for each outcome are shown in Fig 2.

**Table 3:** Hazard Ratio for Outcomes

Outcome	Unadjusted Model		Model 1^a^		Model 2^b^		Model 3^c^	
	HR (95% CI)	*P*	HR (95% CI)	*P*	HR (95% CI)	*P*	HR (95% CI)	*P*
Kidney outcome	0.78 (0.75-0.82)	<0.001	0.84 (0.80-0.88)	<0.001	0.85 (0.82-0.89)	<0.001	0.83 (0.80-0.87)	<0.001
Mortality without kidney outcome	0.60 (0.54-0.66)	<0.001	0.51 (0.46-0.58)	<0.001	0.57 (0.52-0.64)	<0.001	0.56 (0.51-0.63)	<0.001
Mortality^d^	0.83 (0.77-0.90)	<0.001	0.68 (0.63-0.74)	<0.001	0.78 (0.72-0.85)	<0.001	0.78 (0.71-0.84)	<0.001

Abbreviations: CI, confidence interval; HR, hazard ratio.

a Variables used for weighting: age, sex, and weight.

b Variables used for weighting: age, sex, weight, hypertension, diabetes, hypercholesterolemia, and chronic kidney disease.

c Variables used for weighting: age, sex, weight, hypertension, diabetes, hypercholesterolemia, chronic kidney disease, myocardial infarct, congestive heart failure, cerebrovascular disease, and peripheral vascular disease.

d Additionally adjusted variables: systolic blood pressure, diastolic blood pressure, heart rate, peripheral oxygen saturation, fraction of inspired oxygen, mechanical ventilation, and estimated glomerular filtration rate.

### Sensitivity Analysis

Sensitivity analyses were concordant with the primary findings (Tables S1-S4). Using stabilized ATE weighting in place of overlap weighting, statin prescription remained associated with lower risk of the kidney outcome, mortality without prior kidney outcome, and overall mortality, with hazard ratios of 0.81 (95% CI, 0.77-0.85), 0.62 (95% CI, 0.55-0.71), and 0.78 (95% CI, 0.71-0.84), respectively (Table S1). Fine–Gray subdistribution models with overlap weighting yielded consistent estimates—0.83 (95% CI, 0.79-0.87) for the kidney outcome and 0.59 (95% CI, 0.52-0.67) for mortality (Table S2). Redefining exposure as any statin received within 7 days before sepsis onset produced similar results: 0.85 (95% CI, 0.82-0.89), 0.61 (95% CI, 0.55-0.69), and 0.77 (95% CI, 0.72-0.84) for the 3 outcomes, respectively (Table S3). In a negative control analysis using oral laxatives, both the control only and neither medication groups showed higher risks than the statin group across outcomes (Table S4).

### Subgroup Analysis

**Figure 3:**
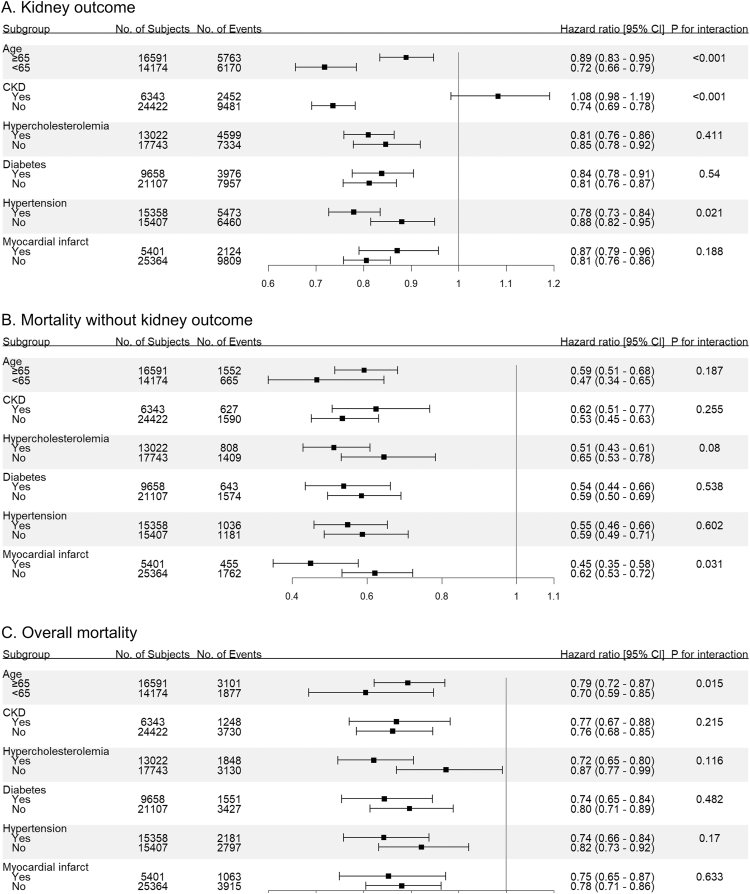
Forest plot of subgroup analysis for hazard ratio for outcomes. (A) Kidney outcome. (B) Mortality without kidney outcome. (C) Overall mortality. Abbreviations: CI, confidence interval; CKD, chronic kidney disease.

Subgroup findings were broadly consistent across prespecified strata (Fig 3). For the kidney outcome, statin prescription was associated with lower risk across most subgroups. For death without prior kidney outcome and for overall mortality, risk reductions were directionally consistent across all subgroups. Tests for interaction indicated heterogeneity: the association with the kidney outcome was stronger among patients aged <65 years, those without CKD, and those with hypertension (*P* for interaction <0.05 for each); for death without prior kidney outcome, the association was stronger among patients with a history of myocardial infarction (*P* for interaction <0.05).

## Discussion

In this cohort, statin exposure before the onset of sepsis was associated with lower risks of the kidney outcome, death without prior kidney outcome, and all-cause mortality. These associations remained significant across sensitivity analyses, with presepsis statin prescription consistently associated with lower risks of the kidney outcome and mortality. Subgroup analyses were broadly consistent across prespecified strata. Tests for interaction indicated modest heterogeneity for the kidney outcome, with stronger risk reductions among patients younger than 65 years, those without CKD, and those with hypertension. For death without kidney outcome, the protective association was amplified in patients with a history of myocardial infarction.

The differences between the results of this study and existing literature may be largely attributed to variations in sepsis definitions, as many prior studies were conducted before the introduction of the Sepsis-3 criteria, resulting in inconsistent definitions of sepsis. For instance, a meta-analysis evaluating the effect of statins on mortality in patients with sepsis used Sepsis-1, which may have contributed to the lack of significant findings compared with our study.^16^ Additionally, a previous study reporting an increased risk associated with statin prescription had small sample sizes, which may have limited the robustness of their analyses.^39^ Another reason for discrepancies with previous studies may involve differences in study design and adjustment variables. Some prior studies may have used a limited set of covariates to evaluate the effects of statins in sepsis, potentially leaving confounding factors insufficiently controlled. To address these issues, this study employed the overlap weighting technique and performed sensitivity analyses, enhancing the reliability of the findings. These methods enhanced confounding control and enabled more precise estimation of the association between presepsis statin prescription and risks of kidney outcome and mortality.

Sepsis-induced AKI is primarily driven by excessive inflammatory responses. When the immune system in patients with sepsis recognizes pathogens, it releases large amounts of proinflammatory cytokines (eg, IL-1β, IL-6, and tumor necrosis factor-α), which damage kidney cells and can lead to AKI.^40^ This overactivation of immune responses is known to accelerate AKI by causing endothelial dysfunction and increasing oxidative stress within the vasculature.^41^^,^^42^ Moreover, sepsis can lead to death not only through the spread of infection but also through excessive systemic inflammatory responses, often referred to as a cytokine storm.^43^ This excessive inflammation increases vascular permeability, which can cause ischemic injury to major organs, leading to multiorgan failure and elevated mortality rates in patients with sepsis.^44^ In this context, statins are thought to improve outcomes in patients with sepsis by inhibiting inflammatory responses and protecting endothelial function, which reduces cytokine release and oxidative stress. Research studies indicate that statins decrease levels of inflammatory cytokines such as IL-6, helping to mitigate kidney damage and systemic inflammation in sepsis.^45^, ^46^, ^47^, ^48^, ^49^ These mechanisms suggest that statins may have a protective effect against kidney injury and reduce mortality in patients with sepsis.

In the subgroup analysis, statin administration was associated with a significant reduction in all outcomes, including among patients without hypercholesterolemia. This supports potential clinical benefits of statins in sepsis irrespective of hypercholesterolemia status and argues for consideration of their prescription in such patients. The attenuated association with the kidney outcome observed in older adults and in patients with CKD may reflect differential competing risk—these strata have higher early mortality, which can preclude the occurrence or ascertainment of the kidney endpoint—and reduced sensitivity of the endpoint in advanced CKD, given that creatinine doubling is nonlinear with respect to GFR and becomes less frequently observed at lower baseline GFR. Moreover, older adults and patients with CKD are high-risk groups for AKI in whom kidneys are intrinsically vulnerable and may not be sufficiently protected by statin therapy alone.^50^^,^^51^ Finally, the stronger association among patients with hypertension is biologically plausible, as endothelial dysfunction and microvascular abnormalities characteristic of hypertension may heighten responsiveness to statins’ pleiotropic, endothelial-stabilizing effects, thereby amplifying benefit.^52^

Overlap weighting was deliberately chosen over conventional inverse probability of treatment weighting because it directs inference toward the subgroup in which the decision to prescribe statins is most clinically uncertain—namely, those with propensity scores near 0.5.^25^ Unlike inverse probability of treatment weighting, which may assign disproportionately large weights to individuals with very low or very high propensity scores and thus estimate an ATE across the entire cohort, overlap weighting produces a set of stable, bounded weights that naturally down-weight the extremes and estimate the ATE within the overlap population.^53^ By concentrating on this equipoise cohort—patients for whom the benefit of statin therapy is most ambiguous—our cause-specific hazard ratios more precisely reflect the effect of statins in the very group for whom treatment decisions are most challenging. Observing a significantly lower risk of AKI and mortality in this equivocal subgroup provides preliminary support for extending statin therapy to patients whose baseline characteristics would otherwise leave clinicians undecided. We believe this approach enhances the clinical relevance of our findings by aligning the estimand with the real-world contexts in which treatment decisions are made.

This study has certain limitations inherent to its retrospective design, including the inability to fully exclude potential confounding variables and residual confounding. Although overlap weighting was used to adjust for confounding as much as possible, the possibility of residual confounding may still remain. In particular, several clinically relevant confounders were incompletely captured—such as source of infection and exposure to nephrotoxins or renin angiotensin system inhibitors—which could bias effect estimates. Medication granularity was limited; we could not reliably distinguish effects by statin agent, lipophilicity, or intensity, nor evaluate dose–response, and thus cannot infer whether benefits vary across statin types or dosing regimens. In addition, we could not ascertain inpatient statin orders or confirm actual in-hospital administration, and some exposure misclassification cannot be excluded. The single-center setting may also constrain generalizability because practice patterns, formularies, and sepsis case mix can differ elsewhere. Further validation in different populations is necessary to determine the reproducibility of these results. To more clearly evaluate the effects of statins in patients with sepsis, a large-scale randomized controlled trial with sepsis patients defined by Sepsis-3 criteria is warranted.

In conclusion, this study demonstrated that statin prescription in patients with sepsis may have beneficial effects on reducing the risks of kidney injury and mortality. Specifically, statin prescription was associated with a decreased risk of kidney injury and a reduction in mortality regardless of whether kidney injury occurred. Furthermore, the benefits of statin prescription were observed irrespective of hypercholesterolemia status, suggesting potential clinical advantages for a broader range of patients with sepsis. However, as this is a single-center retrospective observational study, additional large-scale, multicenter studies and randomized controlled trials are needed to confirm the applicability of these findings to broader patient populations.
