# Supplementary material for: Association of Presepsis Statin Prescription With Kidney and Mortality Outcomes: Cause-Specific, Overlap-Weighted Analyses
Source: Kidney Med. 2026 May 8;8(7):101397. doi: 10.1016/j.xkme.2026.101397 (PMC13241848; doi:10.1016/j.xkme.2026.101397)

Association of Pre-Sepsis Statin Prescription With Kidney and Mortality Outcomes: Cause-Specific, Overlap-Weighted Analyses

Min Woo Kang, Soojeong Yun, Seung Min Song, Ji Eun Kim, Hyo Jin Kim, Eun Jung Cho, Young Joo Kwon, and Shin Young Ahn

Supplementary Table S1-S4

Supplementary Figure S1-S2

Table S1. Hazard ratios from stabilized average treatment effect–weighted cause-specific cox model

Outcome	Hazard ratio	p
**Kidney outcome**	0.81 (0.77 - 0.85)[a]	<0.001
**Mortality without kidney outcome**	0.62 (0.55 - 0.71)[a]	<0.001
**Mortality**	0.78 (0.71 - 0.84)[b]	<0.001

a: Variables used for weight: age, sex, weight, hypertension, diabetes, hypercholesterolemia, chronic kidney disease, myocardial infarct, congestive heart failure, cerebrovascular disease, peripheral vascular disease.

b: Additionally adjusted variables: systolic blood pressure, diastolic blood pressure, heart rate, peripheral oxygen saturation, fraction of inspired oxygen, mechanical ventilation, estimated glomerular filtration rate.

Table S2. Hazard ratios from Fine–Gray subdistribution models

Outcome	Hazard ratio	p
**Kidney outcome**	0.83 (0.79–0.87)[a]	<0.001
**Mortality**	0.59 (0.52–0.67)[a]	<0.001

a: Variables used for weight: age, sex, weight, hypertension, diabetes, hypercholesterolemia, chronic kidney disease, myocardial infarct, congestive heart failure, cerebrovascular disease, peripheral vascular disease.

Table S3. Hazard ratios for outcomes by 7-day pre-sepsis statin exposure

Outcome	Hazard ratio	p
**Kidney outcome**	0.85 (0.82 - 0.89)[a]	<0.001
**Mortality without kidney outcome**	0.61 (0.55 - 0.69)[a]	<0.001
**Mortality**	0.77 (0.72 - 0.84)[b]	<0.001

a: Variables used for weight: age, sex, weight, hypertension, diabetes, hypercholesterolemia, chronic kidney disease, myocardial infarct, congestive heart failure, cerebrovascular disease, peripheral vascular disease.

b: Additionally adjusted variables: systolic blood pressure, diastolic blood pressure, heart rate, peripheral oxygen saturation, fraction of inspired oxygen, mechanical ventilation, estimated glomerular filtration rate.

Table S4. Hazard ratio for outcomes by statin prescription and control oral medication

Outcome	Exposure group	Hazard ratio	p
**Kidney outcome**	Statin	1 (reference)	
	Control-only oral medication	1.25 (1.15 - 1.37)[a]	<0.001
	Neither statin nor control oral medication	1.35 (1.24 - 1.47)[a]	<0.001
**Mortality without kidney outcome**	Statin	1 (reference)	
	Control-only oral medication	1.42 (1.16–1.74)[a]	<0.001
	Neither statin nor control oral medication	1.43 (1.17–1.75)[a]	<0.001
**Mortality**	Statin	1 (reference)	
	Control-only oral medication	1.21 (1.06 - 1.38)[b]	0.002
	Neither statin nor control oral medication	1.20 (1.05 - 1.37)[b]	<0.001

a: Variables used for weight: age, sex, weight, hypertension, diabetes, hypercholesterolemia, chronic kidney disease, myocardial infarct, congestive heart failure, cerebrovascular disease, peripheral vascular disease.

b: Additionally adjusted variables: systolic blood pressure, diastolic blood pressure, heart rate, peripheral oxygen saturation, fraction of inspired oxygen, mechanical ventilation, estimated glomerular filtration rate.

Figure S1. Absolute standardized mean differences for the 11 covariates with the largest baseline imbalance

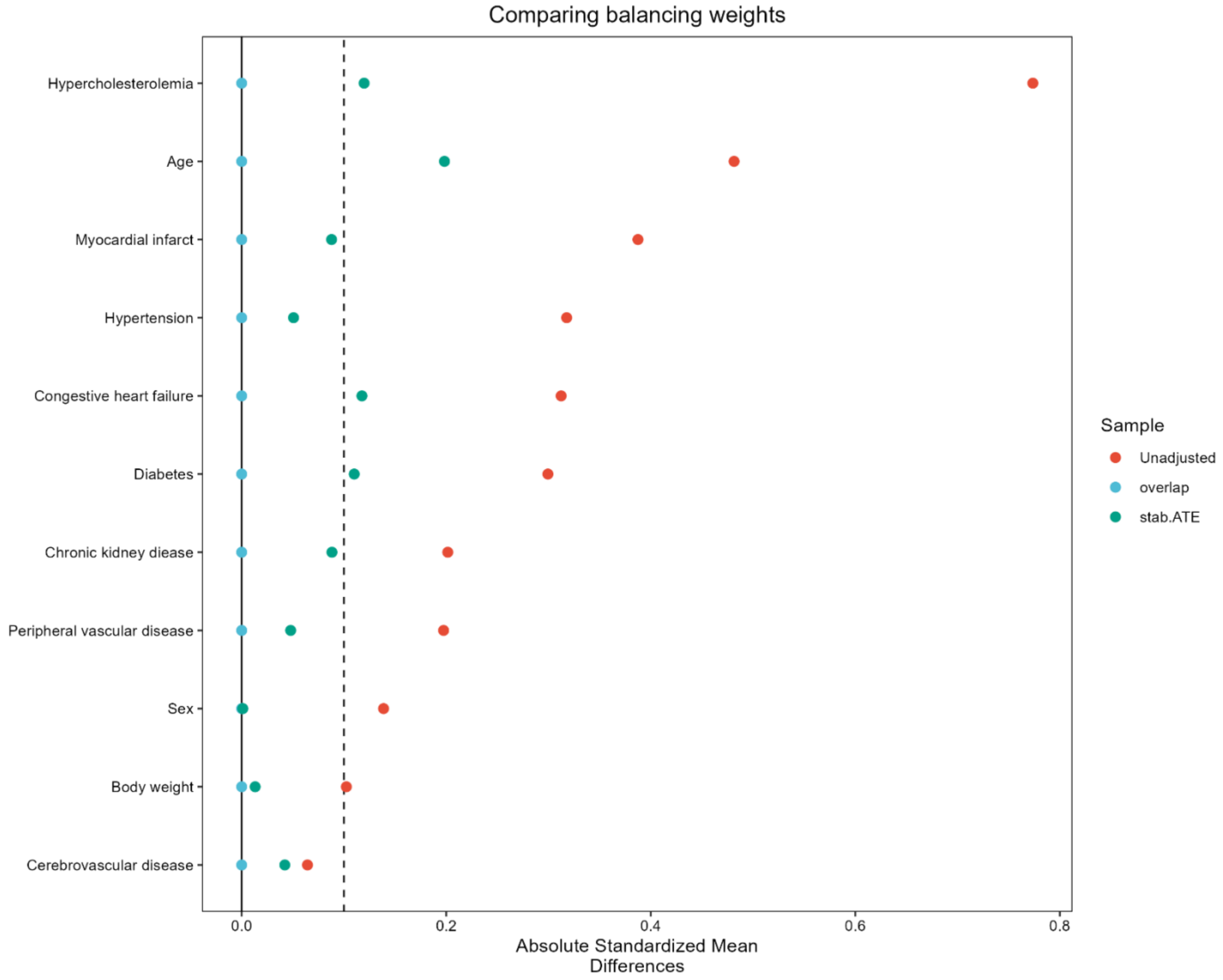

Abbreviations: stab.ATE, stabilized average treatment effect

Figure S2. Comparing distributions of age and hypercholesterolemia across statin prescription and not prescription

A. Unadjusted

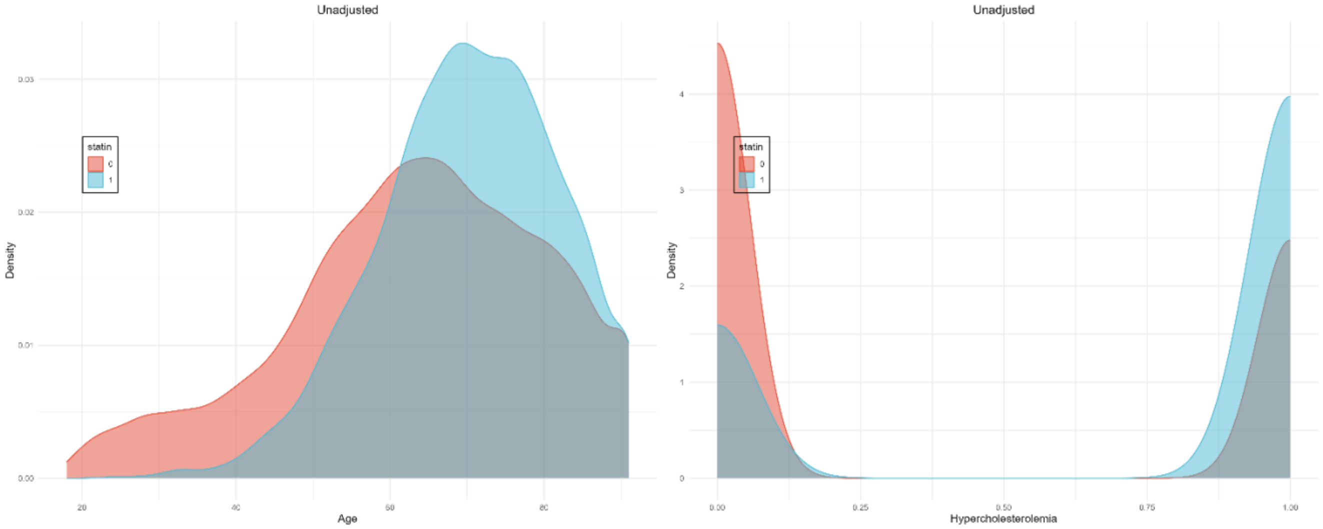

B. Overlap weights

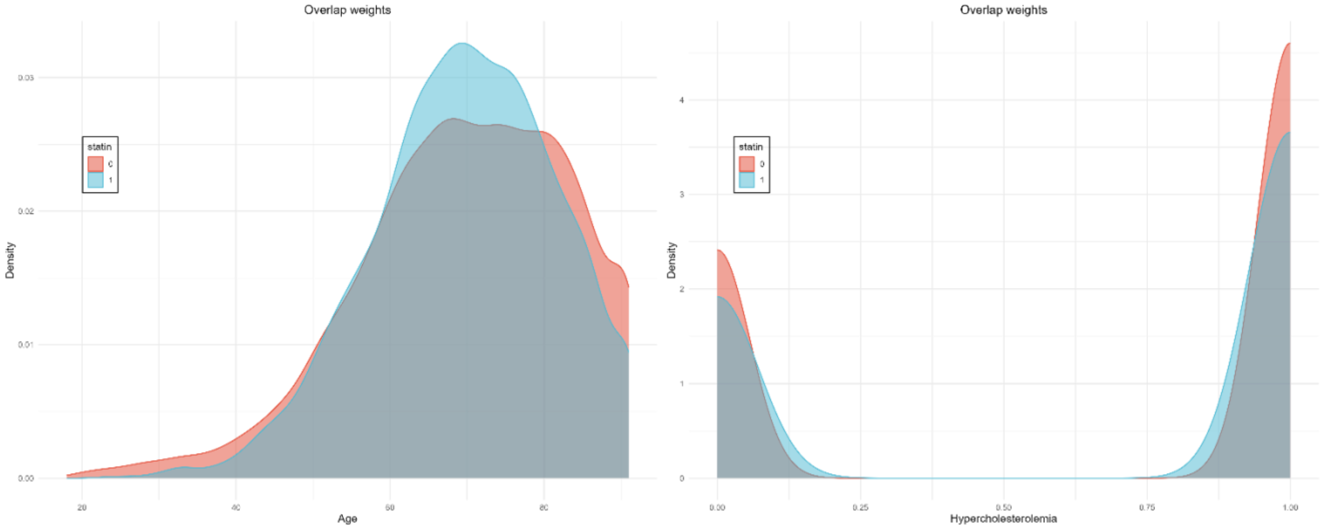

C. Stabilized average treatment effect weights

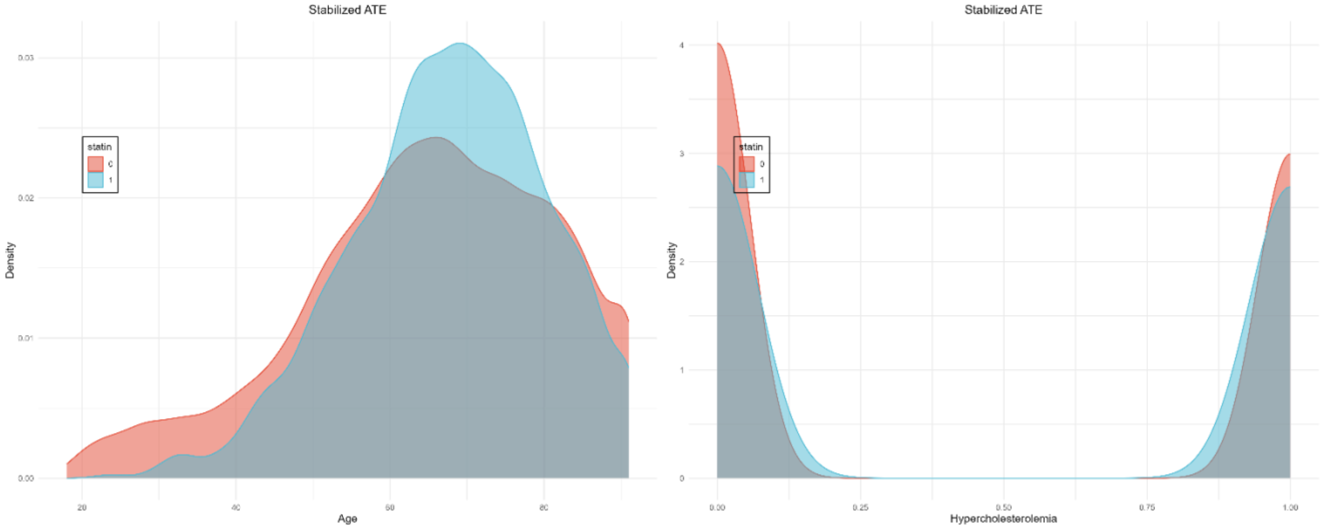

Supplement: Supplementary File (PDF) — Figure S1 and S2; Table S1-S4 [file mmc1.pdf]
